# Various pAQU plasmids possibly contribute to disseminate tetracycline resistance gene *tet*(M) among marine bacterial community

**DOI:** 10.3389/fmicb.2014.00152

**Published:** 2014-05-12

**Authors:** Lisa Nonaka, Fumito Maruyama, Yuki Onishi, Takeshi Kobayashi, Yoshitoshi Ogura, Tetsuya Hayashi, Satoru Suzuki, Michiaki Masuda

**Affiliations:** ^1^Department of Microbiology, Dokkyo Medical University School of MedicineMibu, Tochigi, Japan; ^2^Graduate School of Medical and Dental Sciences, Tokyo Medical and Dental UniversityBunkyo-ku, Tokyo, Japan; ^3^Division of Aquatic Biosciences, Center for Marine Environmental Studies, Ehime UniversityMatsuyama, Ehime, Japan; ^4^Department of Ophthalmology and Regenerative Medicine, Ehime University Graduate School of MedicineShitsukawa, Toon, Ehime, Japan; ^5^Division of Microbial Genomics, Department of Genomics and Bioenvironmental Science, Frontier Science Research Centre, University of MiyazakiKiyotake, Miyazaki, Japan

**Keywords:** pAQU group, pAQU2, transferable plasmid, *tet*(M), antimicrobial resistance genes, SXT/R391 ICEs, aquaculture, *traI*

## Abstract

Emergence of antibiotic-resistant bacteria in the aquaculture environment is a significant problem for disease control of cultured fish as well as in human public health. Conjugative mobile genetic elements (MGEs) are involved in dissemination of antibiotic resistance genes (ARGs) among marine bacteria. In the present study, we first designed a PCR targeting *traI* gene encoding essential relaxase for conjugation. By this new PCR, we demonstrated that five of 83 strains isolated from a coastal aquaculture site had *traI-positive* MGEs. While one of the five strains that belonged to *Shewanella* sp*.* was shown to have an integrative conjugative element of the SXT/R391 family (ICE*Vch*Mex-like), the MGEs of the other four strains of *Vibrio* spp. were shown to have the backbone structure similar to that of previously described in pAQU1. The backbone structure shared by the pAQU1-like plasmids in the four strains corresponded to a ~100-kbp highly conserved region required for replication, partition and conjugative transfer, suggesting that these plasmids constituted “pAQU group.” The pAQU group plasmids were shown to be capable of conjugative transfer of *tet*(M) and other ARGs from the *Vibrio* strains to *E*. *coli*. The pAQU group plasmid in one of the examined strains was designated as pAQU2, and its complete nucleotide sequence was determined and compared with that of pAQU1. The results revealed that pAQU2 contained fewer ARGs than pAQU1 did, and most of the ARGs in both of these plasmids were located in the similar region where multiple transposases were found, suggesting that the ARGs were introduced by several events of DNA transposition into an ancestral plasmid followed by drug selection in the aquaculture site. The results of the present study indicate that the “pAQU group” plasmids may play an important role in dissemination of ARGs in the marine environment.

## Introduction

Wide ranges of antimicrobial agents have been used not only for treatment of human infectious diseases but also in the fish farming industry for therapeutic and prophylactic purposes (Cabello et al., 2013). Among these, tetracyclines (TCs) are broad-spectrum agents, exhibiting activity against various microorganisms, such as Gram-positive and -negative bacteria, chlamydiae, mycoplasmas, and rickettsiae. In some countries, TCs are added to animal feeds at subtherapeutic levels so that they can act as growth promoters (Chopra and Roberts, 2001). In Japan, oxytetracyline and doxycyline have been included in the drugs permitted to use in the process of aquaculture, and the former is one of the drugs available to use for the largest variety of fish species (Ministry of Agriculture, Forestry and Fisheries, Japan, 2011). When the antibiotics are used in the aquaculture, they are usually mixed with feed and administered to fish, and the extensive use of TCs has led emergence of resistant bacteria (Nonaka et al., 2007). To date, 46 different TC resistance genes have been submitted to the nomenclature center (http://faculty.washington.edu/marilynr/), of which 30 genes code for efflux proteins, 12 genes for ribosomal protection proteins and four genes for other categories. Emergence and dissemination of drug resistance in the marine environment have a large impact on the fish farming industry. It is also an important issue for clinical medicine because a number of antibiotics resistance genes (ARGs) are shared between marine bacteria and human pathogens (Cabello et al., 2013) and the gene flow between these environments has been suggested. Further, transfer of opportunistic human pathogen from aquatic environments possible to generate antimicrobial resistance infections (Cabello et al., 2013).

A TC resistance gene, *tet*(M), is among the ARGs shared between marine bacteria and human pathogens and has extensively been studied in terms of the molecular ecology in the environment. The gene is known to be distributed among numerous bacterial species (Chopra and Roberts, 2001; Roberts, 2005) derived from diverse sources, such as human pathogens (Jones et al., 2006), domestic animals (Aminov et al., 2001; Chee-Sanford et al., 2001), food (Gevers et al., 2003; Bertrand et al., 2005), and farmed fish (Kim et al., 2007). In our previous study (Kim et al., 2004; Nonaka et al., 2007), we also found that *tet*(M) was distributed among several bacterial species isolated from fishes or the marine aquaculture environment. Other recent studies have also reported identification of *tet*(M) in the bacteria from the aquaculture environments (Nikolakopoulou et al., 2008; Seyfried et al., 2010; Di Cesare et al., 2012, 2013). Thus, the marine bacteria have been suggested to be a reservoir of *tet*(M).

While detailed mechanisms by which *tet*(M) has propagated in the marine environment are unknown, it has previously been demonstrated that *tet*(M) is frequently carried by mobile genetic elements (MGEs), such as plasmid and transposon (Chopra and Roberts, 2001). Previously, 73 strains of *tet*(M)-positive marine bacteria from an aquaculture site were examined, and it was shown that five of them, strains 04Ya001, 04Ya016, 04Ya090, 04Ya108, and 04Ya311, were able to transfer TC resistance to *E*. *coli* (Neela et al., 2009). Among these strains, 04Ya311 was characterized in detail, and it was demonstrated that this strain had a novel self-transferable plasmid, which we designated as pAQU1, and that conjugative transfer of pAQU1 conferred TC resistance on *E. coli* (Nonaka et al., 2012). Determination of its complete nucleotide sequence revealed that pAQU1 encoded *tet*(M) and other ARGs, such as *tet*(B), *sul*2, *flo*R and genes similar to *bla*_CARB−9_, *mph*(A) and *mef*(A). Phylogenetic analysis of pAQU1's *traI* gene encoding relaxase (Nonaka et al., 2012), an only protein component of the conjugative machinery common to all MGEs using a conjugative system (Garcillan-Barcia et al., 2009; Smillie et al., 2010), indicated that the plasmid had a close relationship with IncA/C plasmid and SXT/R391 family of integrative conjugative elements (SXT/R391 ICEs) which belong to the MOB_H12_ clade in the MOB_H_ family (Nonaka et al., 2012). Since IncA/C plasmid and SXT/R391 ICEs are distributed among many aquatic isolates of genera *Enterobacteriaceae* and *Vibrionaceae* (Welch et al., 2007; McIntosh et al., 2008; Fricke et al., 2009), we hypothesized that pAQU1 found in 04Ya311 was a novel plasmid that might play a role in dissemination of *tet*(M) in the marine environment. In present study, since several pAQU1-related plasmids were found, we aimed to reveal that these pAQU1- related plasmids constituted a novel “pAQU group” characterized by a common backbone structure and conferred to transfer *tet*(M) and other ARGs to *E. coli* by conjugation.

## Materials and methods

### Bacterial strains

For screening of *traI*, 83 tetracycline-resistant strains of bacteria isolated from the sediment or seawater of a coastal aquaculture site in Japan were used (Supplementary Table 1). At the site, oxytetracycline and flumequine had been used (Nonaka et al., 2007). Theses environmental strains were cultured at 25°C in the brain heart infusion (BHI) medium (Becton, Dickinson and Company, Sparks, MD, USA) with 0.2% NaCl and 10 μg/ml of TC (Nakalai tesque, Kyoto, Japan). All of the strains were confirmed by PCR to have *tet*(M) (Nonaka et al., 2007), and strains 04Ya001, 04Ya016, 04Ya090, and 04Ya108 have previously been shown to transfer *tet*(M) to *E. coli* (Neela et al., 2009). *E. coli* W3110 obtained from the National BioResource Project (National Institute of Genetics, Japan) and its rifampicin-resistant derivative, W3110Rif^r^ (Nonaka et al., 2012), were cultured at 37°C in the Luria-Bertani medium (BD) with or without 10 μg/ml (for liquid media) or 20 μg/ml (solid media) of TC and 100 μg/ml of rifampicin (Sigma-Aldrich, Saint-Louis, MO).

### DNA extraction and PCR

Total bacterial DNA was extracted by using a QuickGene DNA tissue kit (Fujifilm Corporation, Tokyo, Japan) according to the manufacturer's protocol.

A set of degenerate primers (MOBH1_traI062-1F, MOBH1_traI062-2R) listed in Table 1 were designed and used for detecting *traI* gene of MGEs of MOB_H12_ clade of MOB_H_ family. Namely, the *traI* nucleotide sequences of pAQU1 (GenBank accession number AB571865), IncA/C plasmids including pYR (CP000602), pIP1202 (CP000603), pP99-018 (AB277723), pP91278 (AB277724), pSN254 (CP000604) (Welch et al., 2007; Fricke et al., 2009), SXT/R391 ICEs including ICE*Vch*Mex (DQ180350, GQ463143), ICE*Vch*Ind4 (GQ463141), ICE*Vch*Ind5 (GQ463142), ICE*Vch*Ban5 (GQ463140), ICE*Pal*Ban1 (GQ463139), SXT^MO10^ (AY090559), ICE*Pda*Spa1 (AJ870986), and R391(AY090559) (Wozniak et al., 2009) were aligned using the multiple-sequence alignment program Clustal W (Thompson et al., 1994; Larkin et al., 2007) and conserved regions were selected for the designing of the primers. The PCR mixture (total volume, 10 μ l) contained 5 μ M each of the primers, 1 × PCR buffer (Life Technologies Corporation, Carlsbad, CA, USA), 2.5 mM each of dNTPs, 0.5 U of AmpliTaq DNA polymerase (Life Technologies Corporation) and the template DNA (20–50 ng). PCR amplification was performed with initial denaturation at 94°C for 1 min followed by 30 cycles of 94°C for 30 s, 57°C for 30 s, and 72°C for 30 s and a final extension step at 72°C for 3 min.

**Table 1:** **PCR primers used in this study**.

**Gene (s)**	**MGE (s)**	**Primer name**	**Primer sequence (5′ -> 3′)**	**Annealing temperature (°C)**	**Size (bp)**	**References**
*traI*	pAQU1 (GenBank accession number AB571865)	MOBH1_traI062-1F MOBH1_traI062-2R	CAYYTRYTRCCAGCWTCYGA SRCGYCWSGCTSATTGAC	57	645	This study
	IncA/C plasmids^*^					
	SXT/R391 family of ICEs^**^					
*tet*(B)	pAQU1 (AB571865)	TetB-FW	TACGTGAATTTATTGCTTCGG	65	226	This study
		TetB-RV	ATACAGCATCCAAAGCGCAC			
*tet*(M)		tet(M)-1	GTTAAATAGTGTTCTTGGAG	55	676	Nonaka et al., 2007
		tet(M)-2	CTAAGATATGGCTCTAACAA			
*bla*_CARB-9_-like		cds084-1f	ATGAGTACATTCAAGACATT	55	672	This study
		cds084-672r	TTATTTGACAGCATACTCTT			
*mph*(A)-like		cds165-1f	ATGAAAAATAGAGATATTCA	55	885	This study
		cds165-885r	CTACTCAACACCTAACTGTA			
*mef*(A)-like		cds166-1f	ATGGAAAACCGTAAATGGTT	55	1,224	This study
		cds166-1124r	TTAAATATTTTTGATTTTAC			
*sul*2		sul2-F	CTCAATGATATTCGCGGTTTYCC	58	245	Heuer and Smalla, 2007
		sul2-R	AAAAACCCCATGCCGGGRTC			
*flo*R		FloR283f	GGAGCAGCTTGGTCTTCAAC	55	226	This study
		FloR519r	AATGAATATCGCCTGCCATC			
*repA*		CDS001(AB571865)-446f	GGAAGAAGCATCCAGAGCAC	55	236	This study
		CDS001(AB571865)-646r	AGCCAGAGACGCTGTTGATT			
*traA2*		traA89f	AAGCTCACGCTGGTACTGGT	67	177	This study
		traA246r	CATTGCGAATGCCATGATAC			
*traB*		traB343f	CGCGATGTTAAGGAGCTTTC	55	268	This study
		traB591r	CTTACCCGTTGGTTTGCTGT			
*traC*		traC1742f	CCGCGTATACCGCTTATGTT	55	388	This study
		traC2110r	CAGCAGGATCGTCAAAGTGA			
*traF*		traF1f	GTGGGATAACCCAACCATTG	55	298	This study
		traF1r	GGAGCCTGTATTTCGCACAT			
*traN*		traN535f	CCAGGCGATGTGGTTAGTTT	67	1,085	This study
		traN1600r	CTTTGAGAGCCTGAATTGCC			
*traI*		traI1976f	ATGTTGATGTGCGTGGTCAT	55	270	This study
		traI2225r	CTTCCGGTTTGGATGAACTG			
*traL*		TraL70f	CTGGCTCCTATGCTTCTTGG	55	220	This study
		TraL270r	CCTCCGGATAAATGGGTTTT			
*Int*	ICE*Vch*Mex (DQ180350)	int575f	TCACAGCAAAGAAAGTCGGC	57	229	This study
		int803r	CGGATCTCTATGGGCACTGT			
*traF*		traF517f	CGTTCGGATTGCCCTTACTG	57	192	This study
		traF708r	AACTAAGAACACGGCAGGGA			
*traN*		traN1087f	ATTCAATGCACGGATCAGCC	57	202	This study
		traN1288r	CACAGGAAGGATTCGCTTCG			

* IncA/C plasmid including pYR (CP000602), pIP1202 (CP000603), pP99-018 (AB277723), pP91278 (AB277724), pSN254 (CP000604) (Welch et al., 2007; Fricke et al., 2009).

** SXT/R391 ICEs including ICEVchMex (DQ180350, GQ463143), ICEVchInd4 (GQ463141), ICEVchInd5 (GQ463142), ICEVchBan5 (GQ463140), ICEPalBan1 (GQ463139), SXT^MO10^ (AY090559), ICEPdaSpa1 (AJ870986) and R391(AY090559) (Wozniak et al., 2009).

For PCR detection of ARGs [*tet*(B), *tet*(M), *sul*2, *flo*R, *bla*_CARB−9_-like, *mph*(A)-like and *mef*(A)-like], genes related to the type IV secretion system (*repA, traA2, traB, traC, traF, traN, traI*, and *traL)* and ICE*Vch*Mex-related genes (*int, traF*, and *traN*), respective primers shown in Table 1 were used. PCR amplification was performed with initial denaturation at 94°C for 1 min followed by 30 cycles of denaturation at 94°C for 30 s, 1 min of annealing at the temperatures shown in Table 1, and extension at 72°C for 1 min and a final extension step at 72°C for 3 min.

Additional 12 sets of primers (Table 2) were designed for PCR to identify and characterize the backbone structure of the “pAQU group” plasmids. The PCR mixture (total volume, 10 μ l) contained 0.4 μ M each of the primers, 1 × LA PCR buffer II (TAKARA BIO INC, Ohtsu, Japan), 0.4 mM each of dNTPs, 2.5 mM of MgCl_2_, 0.5 U of LA Taq DNA polymerase (TAKARA BIO INC) and the template DNA (20–50 ng). For amplicons of 2–10 kb, PCR amplification was performed with initial denaturation at 94°C for 1 min followed by 30 cycles of denaturation at 94°C for 30 s, annealing at 55°C for 1 min, and extension at 72°C for 5 min and a final extension step at 72°C for 3 min. For amplicons longer than 10 kb, two-step PCR was performed with 30 cycles of denaturation at 96°C for 20 s and extension at 69°C for 16 min.

**Table 2:** **PCR primers for detection of the conserved region between pAQU1 and pAQU2 (pAQU backbone)**.

**Region number**	**Primer name**	**Primer sequence (5′ -> 3′)**	**Product size (bp)**	**Relevant genes contained in the amplicon**
1	AB571865-1	ATGACAACAACACTCCACAATGAGG	12,606	*rep*A
	170-16f	GCCGCAACGATGACAAACTTAACGA		
2	AB571865-30966	ACAGTTCACCTAAACATTGATG	3,785	*parA, parB*
	AB571865-34750	ACATACAACCAGAAACAGAACC		
3	170-11r	GCTTTGGCGTGTTCTCGGTTATGAT	9,017	*traI, traD*
	170-11f	CGCATGGAACACTACACCGCTATAT		
4	170-10r	GGTTGCTTCACTGCTTCCTATCATG	9,128	*S043*, (*traJ*), *traL, traE, traK, traB, traV, traA1, traA2*
	170-10f	CAGCTCGATCCGTTCTTCACCATTA		
5	170-9r	CGCACATTATCTCCAAGCACCGTTT	9,003	*DsbC, traC*
	170-9f	CAGCAACCCAAAGAAGAGCAAGTAC		
6	AB571865-68599	AAGGAAGTACGGTGGAAG	6,975	*trhF, traW, traU, traN*
	AB571865-75573	CAGGATCGTCAAAGTGAC		
7	170-5r	GCCGCAATGGATCGTTATCGTTTCT	9,143	
	170-5f	TGCTCTGCCGACAACATTGGTTTAC		
8	170-4r	ATTGGGTTGAAGGTTGGAAGGAGTG	9,141	
	170-4f	CGGCATACTCAGGTTCTCCTTCTTT		
9	170-3r	GGCTGGTTGTGGCGATATTGATGAT	9,242	
	170-3f	GCCTTCTGCCAAACCTAATACTCCT		
10	170-21r	GCCAAGTTCGGTAAGAGTGAGAGTT	9,003	*ter*
	170-21f	AGCGTTCTCAATGGTTGGGTTATCC		
11	170-20r	CCTCAAGATCCTTTCCCTATGGTTC	9,142	*traF, traH, traG*
	170-20f	CGACTGCCGTAATTTCTTGCTCTAG		
12	170-19r	CGGAAAGATACGAAGACGGATTACC	9,390	
	170-19f	CTCCAACTCCAATGACCTTCAAGAC		

For determination of the phylogenetic position of the strains were identified by the nucleotide sequences of the bacterial 16S rRNA gene which were amplified using primers 9F (5'-GAGTTTGATCCTGGCTCAG-3') and 1510R (5'-GGCTACCTTGTTACGA-3'). PCR reaction was performed as previously described (Nonaka et al., 2007).

### Sequencing of the PCR products

When required, the nucleotide sequences of the PCR products were determined using the dideoxy chain termination method with a Big Dye terminator V 3.1 cycle sequencing kit (Life Technologies Corporation) and an appropriate primer according to the manufacture's protocol following to the purification of the PCR products using the QIAquick PCR purification kit (QIAGEN GmbH, Hilden Germany). For identification of the sequences of 16S rRNA genes, primers 9F, 339F (5' CTCCTACGGGAGGCAGCAG 3'), 785F, (5'-GGATTAGATACCCTGGTAGTC-3'), 1099F (5'-GCAACGAGCGCAACCC-3'), 536R (5'-GTATTACCGCGGCTGCTG-3'), 802R (5'-TACCAGGGTATCTAATCC-3'), 1242R (5'-CCATTGTAGCACGTGT-3') and 1510R were used.

### Filter mating

The donor marine bacteria and recipient (*E. coli* W3110) were cultured to the mid-log phase (OD600 = 0.5–0.8) and mixed with each other (500 μl each) in triplicates. The mixture was then collected onto a sterile 25 mm in a diameter, 0.2 μm-pore size nitrocellulose membrane filter (Merck millipore, Billerica, MA) by using a KG-25 filter holder (Advantec, Tokyo, Japan) and a vacuum pump at 20 kPa. The filter with the collected cells were rinsed by 10 ml of BHI medium with 2% NaCl or LB and placed on the Marine Broth 2216 plate (Becton, Dickinson and Company) for incubation at 25°C for 20 h. Each filter was then transferred into 500 μl of phosphate-buffered saline (PBS) and agitated vigorously with a Vortex mixer to detach the bacteria. The suspension was serially diluted by 10-fold in PBS and plated onto the media. To select for the transconjugants, LB agar plates with 20 μg/ml of TC were used and incubated for 20 h at the temperature (42°C) which inhibits growth of the donors. For counting the total number of donor cells and the transconjugants, the cell suspension was spread on a BHI agar plate with 0.2% NaCl and 10 μg/ml of TC and incubated at 25°C. When the transconjugants were used as donors, *E. coli* W3110Rif^r^ was used as a recipient and 2nd transconjugants were counted on LB agar plates with 20 μg/ml of tetracycline and 100 μg/ml of rifampicin after incubation at 37°C for 20 h. For counting the total number of the donor cells and the transconjugants, LB plates with 20 μg/ml tetracycline were used and incubated at 37°C for 20 h. The number of the donor cells was obtained by subtracting the number of the transconjugants from the total number of the donor cells and the transconjugants. Transfer rate was calculated as a ratio of the number of transconjugants per donor cell.

### Pulsed-field gel electrophoresis (PFGE) and southern hybridization

To determine whether *tet*(M) is carried by the plasmid or integrated in the chromosome, PFGE and Southern hybridization were performed. First, a DNA-containing agar plug of each sample was prepared by the previously reported method (Kijima-Tanaka et al., 2007) with the following modifications. Culture fluid containing about 10^8^ cells was put into a 15-ml centrifuge tube and centrifuged at 9,000 rpm for 10 min. The cell pellet was re-suspended in 120 μl of cell suspension buffer [100 mM Tris-HCl, 100 mM EDTA (pH 8.0)] and mixed with 120 μl of melted 2% InCert® Agarose (Lonza, Basel, Switzerland) and 12 μl of lysozyme (20 mg/ml; Sigma-Aldrich) dissolved in TE buffer [10 mM Tris-HCl, 1 mM EDTA (pH 8.0)]. The mixture was quickly dispensed into the plug mold (Bio-Rad, Hercules, CA). After solidification, the plug was transferred into 500 μl of TE buffer containing 1% SDS and 12 μl of lysozyme (20 mg/ml), and incubated at 37°C for 1 h. Then, the liquid portion in each tube was replaced with 500 μl of lysis buffer [100 mM Tris-HCl, 100 mM EDTA (pH 8.0), 1% N-lauroylsarcosine] and 50 μl of proteinase K (20 mg/ml; TAKARA BIO INC.), and the plug was incubated at 50°C for overnight. On the next day, the plug was washed three times with 500 μl of TE buffer at room temperature for 15 min, pretreated with 1 ml of S1 nuclease buffer [30 mM sodium acetate (pH 4.6), 1 mM zinc acetate, 5%(v/v) glycerol] at room temperature for 30 min, and treated with 10 U of S1 nuclease (Life Technologies Corporation, Carlsbad, CA) in 200 μl of the S1 nuclease buffer at 37°C for 15 min. The plugs was then washed twice with 1 ml of ice-cold high concentration EDTA-Tris [10 mM Tris-HCl, 100 mM EDTA (pH 8.0)]. The plug was cut into half and one of the pieces was embedded in a 1% Seakem® Gold Agarose (Lonza) gel and PFGE was performed on a CHFE MAPPER™ (Bio-Rad) under the conditions described previously (Nonaka et al., 2012). The DNA size standard lambda ladder (Bio-Rad) was used as a size marker. The DNA in the gel was stained with SYBR Gold (Life Technologies Corporation) at the concentration recommended by the manufacturer and visualized under UV light. Subsequent hybridization was performed according to the standard method (Sambrook and Russell, 2001). PCR-amplified DNA fragments of *tet*(M), *traI* in pAQU1 (*traI*-pAQU1), *traI* in ICE*Vch*Mex (*traI*-ICE*Vch*Mex) and *rep* in pAQU1 (*rep*-pAQU1) were labeled with digoxigenin by using the primers shown in Table 1 and a PCR DIG probe synthesis kit (Roche Diagnostics, Mannheim, Germany). Hybridizations were performed at 41°C for overnight.

### Sequencing and annotation

The entire sequence of the total DNA of TJ090W1 was determined by the pyrosequencing method with the Titanium chemistry and the Genome Sequence FLX system (Roche Diagnostics), and a set of data consisting of 225,251 shotgun and 271,612 paired-end (average insert size: 7 kb) sequences with an average length of 684 and 398 nucleotides were generated, respectively. The volume of the data was estimated to cover 60 times of the entire DNA sequence. The plasmid-derived sequences were obtained by subtracting the genomic sequence of *E*. *coli* W3110 from the entire set of data and then assembled *de novo* by using the GS assembler (Newbler 2.8) (Roche Diagnostics), generating 1 scaffold and 19 sets of assembled contigs (over 500 bp), of which five selected contigs were used for assembling the whole structure of the plasmid molecule according to the previously described method (Nonaka et al., 2012). Preliminary identification of protein coding regions in the plasmid was carried out by using MetaGeneAnnotator (Noguchi et al., 2008). Then, the results were subjected to BLAST searches and compared with the sequences listed in the NCBI non-redundant protein database for their length, identity and coverage, and final annotation was manually performed by using In Silico Molecular Cloning Genomics Edition® (In Silico Biology, Inc., Yokohama, Japan) with the threshold *E*-value of 10^−5^.

### Nucleotide sequence accession number

The entire sequence of pAQU2 was deposited in GeneBank/DDBJ/ EMBL databases under the accession no. AB856327.

## Results

### Screening by PCR detection and sequencing of *trai* gene for the marine bacterial strains which harbor *tet*(M)-bearing pAQU1-like plasmid or ICE*Vch*Mex-like MGE

For screening for the bacterial strains carrying the pAQU1-like plasmids, we designed degenerate primers, MOBH1_traI062-1F and MOBH1_traI062-2R, based on the *traI* nucleotide sequences of pAQU1 (GenBank accession number AB571865), five of IncA/C plasmids (Welch et al., 2007; Fricke et al., 2009) and seven of SXT/R391 ICEs (Wozniak et al., 2009) which belong to the MOB_H12_ clade of the MOB_H_ family (Table 1). When 83 *tet*(M)-positive strains of marine bacteria from the aquaculture site were examined, amplified *traI* DNA of 645 base pairs (bps) was detected in strains 04Ya001, 04Ya016, and 04Ya090 (Table 3) that had been known to carry transferable *tet*(M) gene (Neela et al., 2009). The 645-bp *traI* PCR product was also detected in strains 04Ya007 and 04Ya265 whose ability to transfer *tet*(M) had been unclear (Table 3). Although strain 04Ya108 had been shown to conjugatively transfer *tet*(M) to *E*. *coli* (Neela et al., 2009), *traI* was not detected in this strain (Table 3). *traI* was not detected in 77 other strains, either; 66 strains whose *tet*(M) was shown not to be transferable (Neela et al., 2009) and 11 strains that had not been examined before. Therefore, five of 83 strains examined appeared to carry MGE related to pAQU1 or IncA/C plasmid or SXT/R391 ICE. Then, nucleotide sequences of the PCR-amplified 645-bp *traI* DNA of the plasmids in these five strains were determined. The results indicated that the *traI* sequence of four strains (04Ya001, 04Ya016, 04Ya090, and 04Ya265) were identical to that of pAQU1, whereas the sequence of one strain (04Ya007) was distinct from that of pAQU1, but similar to that of ICE*Vch*Mex (97% identity), which belongs to SXT/R391 ICEs found in *V*. *cholerae* isolated from sewage in Mexico (Burrus et al., 2006). The ICE*Vch*Mex-specific *int, traF* and *traN* genes were also detected by PCR in 04Ya007. Therefore, strains 04Ya001, 04Ya016, 04Ya090, and 04Ya265 were shown to have pAQU1 or closely related pAQU1-like plasmids, whereas 04Ya007 likely had another member of the MOB_H12_ clade, ICE*Vch*Mex-like MGE.

**Table 3:** **Strains harboring *traI* gene belonging to the MOBH_12_ clade in the MOB_H_ family on the basis of a PCR screening**.

**Strain ID**	**Species**	**Origin**	**Isolation date**	**TC resistance**	***tet*(M)**	traI^*^	**References**
04Ya001	*Vibrio splendidus*	Sediment	Apr 26/2004	Yes	+	+	Nonaka et al., 2007; Neela et al., 2009
04Ya007	*Shewanella fidelis*	Sediment	Apr 26/2004	Yes	+	+	This study
04Ya016	*Vibrio splendidus*	Sediment	Apr 26/2004	Yes	+	+	Nonaka et al., 2007; Neela et al., 2009
04Ya090	*Vibrio splendidus*	Sediment	May 21/2004	Yes	+	+	Nonaka et al., 2007; Neela et al., 2009
04Ya108	*Vibrio ponticus*	Sediment	May 21/2004	Yes	+	−	Nonaka et al., 2007; Neela et al., 2009
04Ya265	*Photobacterium aphoticum*	Sediment	Sep 24/2004	Yes	+	+	This study
04Ya311	*Photobacterium damselae* subsp. *damselae*	Seawater	Sep 24/2004	Yes	+	+	Nonaka et al., 2007, 2012; Neela et al., 2009
W3110	*E*. *coli*	Laboratory strain	–	No	−	−	Paigen, 1966
W3110Rif^r^	*E*. *coli*	Laboratory strain	–	No	−	−	Nonaka et al., 2012

* Using degenerate primers MOBH1_traI062-1F and MOBH1_traI062-2R in Table 1.

**Figure 1:**
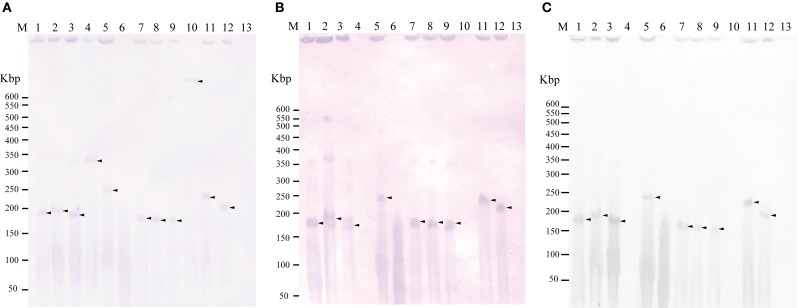
**Detection of plasmid(s) in the donors and transconjugants using Southern hybridization following to pulsed field gel electrophoresis with the *tet*(M) probe (A), *traI*-pAQU1 probe (B), and *rep*-pAQU1 probe (C)**. Lane: M, DNA size standard (lambda ladder); 1, 04Ya001; 2, 04Ya016; 3, 04Ya090; 4, 04Ya108; 5, 04Ya265; 6, 04Ya311; 7, TJ001W1; 8, TJ016W1; 9, TJ090W1; 10, TJ108W1; 11, TJ265W1; 12, TJ311W2 and 13, W3110. Approximately, 20 ng of DNA was loaded in each lane. Arrows indicate the positions of the bands.

**Figure 2:**
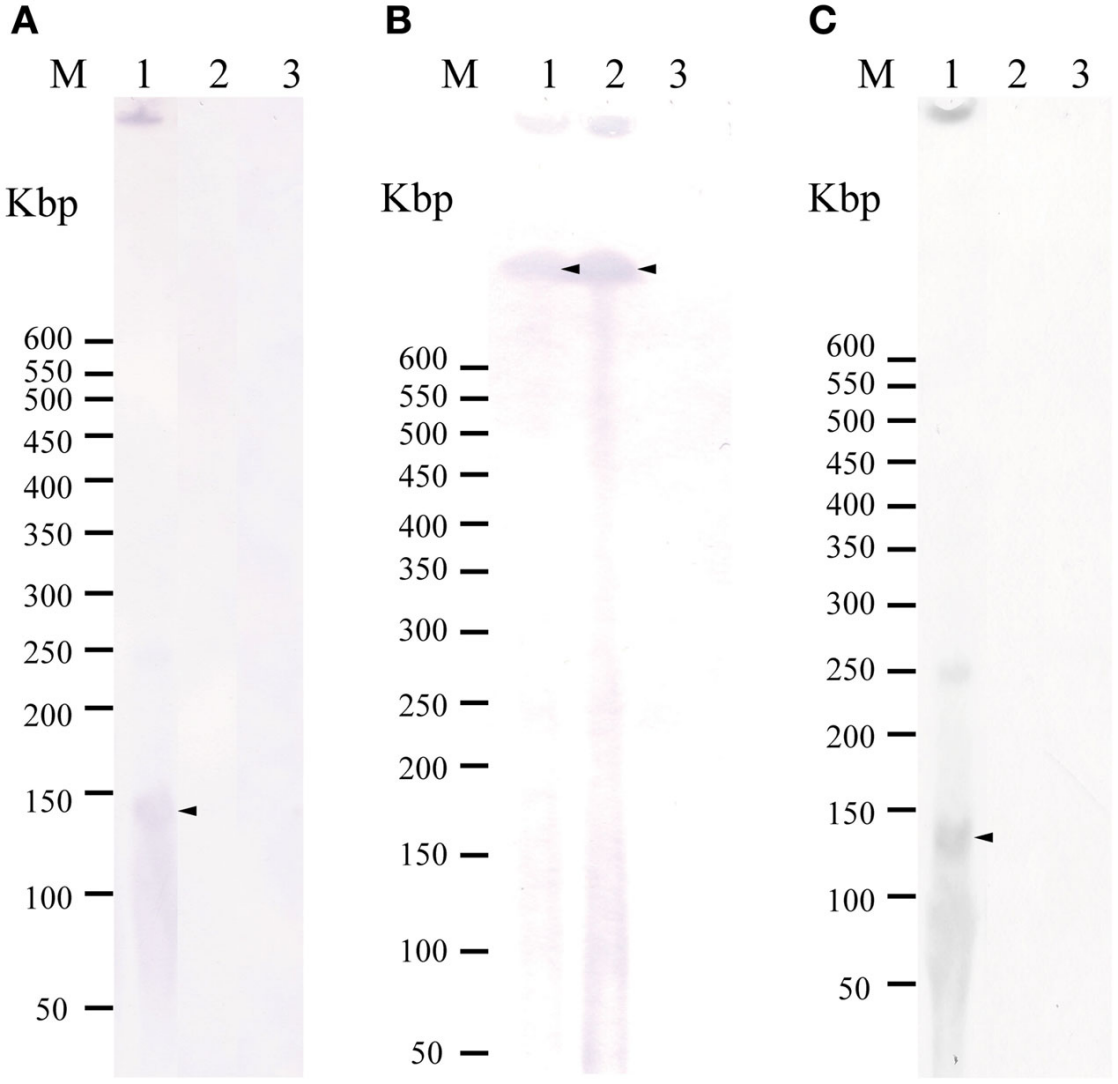
**Detection of a plasmid or ICE(s) in the donors and transconjugants using Southern hybridization following to pulsed field gel electrophoresis with the *tet*(M) probe (A), *traI*-ICE*Vch*Mex probe (B), and *rep*-pAQU1 probe. (C)** Lane: M, DNA size standard (lambda ladder); 1, 04Ya007; 2, TJ007W1; 3, W3110. Approximately, 20 ng of DNA was loaded in each lane. Arrows indicate the positions of the bands.

To examine whether *tet*(M) is also plasmid-borne in strains 04Ya001, 04Ya016, 04Ya090, and 04Ya265 carrying the pAQU1-like plasmids, 04Ya007 with ICE*Vch*Mex-like MGE and 04Ya108 without *traI*, PFGE followed by Southern hybridization was performed. Hybridization with the *tet*(M) probe generated extrachromosomal DNA bands ranging from 150 to 350-kbp in all of the strains examined (Figure 1A, lanes 1–5 and Figure 2A, lane 1). When the *traI*-pAQU1 or *rep*-pAQU1 probe was used, bands of the same sizes were detected for 04Ya001, 04Ya016, 04Ya090, and 04Ya265 (Figures 1B,C, lanes 1–3 and 5), but not 04Ya108 (Figures 1B,C, lane 4) compatible with the PCR results. While when a strain 04Ya007 was used, the *tra*I-ICE*Vch*Mex probe detected a signal at the position corresponding to the chromosomal DNA (Figure 2B, lane 1). The results indicated that in four strains 04Ya001, 04Ya016, 04Ya090, and 04Ya265, *tet*(M) was most likely carried on the pAQU1-like plasmids, whereas in strain 04Ya108, *tet*(M) appeared to be in the plasmid of non-MOB_H12_ clade. In strain 04Ya007, *tet*(M) appeared to be plasmid-borne while ICE*Vch*Mex-like MGE was integrated in the chromosome.

### Conjugative transfer of *tet*(M) gene mediated by the pAQU1-like plasmids

To confirm and examine the ability of the six strains to transfer TC resistance, filter mating experiments were performed by using *E. coli* as a recipient. As reported previously (Neela et al., 2009), strains 04Ya001, 04Ya016, and 04Ya090 were able to transfer TC resistance to *E*. *coli* (Table 4). Strains 04Ya007 and 04Ya265 were also shown to transfer TC resistance to *E. coli* with the frequencies of 1.37 × 10^−4^ and 1.08 × 10^−4^, respectively, at the levels comparable to the positive control strain 04Ya311 (Table 4). Strain 04Ya108, which did not appear to carry MOB_H12_ MGE, was also able to transfer TC resistance, but with a much lower frequency (Table 4).

**Table 4:** **Transfer frequencies of tetracycline resistance by filter mating methods**.

	**Strain**	**Transfer frequencies**
Donors	04Ya001	(1.64 ± 0.56) × 10^−5^
	04Ya007	(1.37 ± 0.18) × 10^−4^
	04Ya016	(1.90 ± 0.73) × 10^−5^
	04Ya090	(7.71 ± 3.84) × 10^−5^
	04Ya108	(1.78 ± 0.16) × 10^−7^
	04Ya265	(1.08 ± 0.18) × 10^−4^
	04Ya311	(4.11 ± 0.84) × 10^−4^
Recipient	*E*. *coli* W3110	
Donors	TJ001W1	(3.90 ± 1.21) × 10^−5^
	TJ007W1	(1.01 ± 0.08) × 10^−3^
	TJ016W1	<(2.12 ± 0.48) × 10^−10^
	TJ090W1	(1.00 ± 0.32) × 10^−4^
	TJ108W1	<(4.88 ± 0.28) × 10^−10^
	TJ265W1	(1.08 ± 1.03) × 10^−4^
	TJ311W2	(6.60 ± 3.01) × 10^−5^
Recipient	*E*. *coli* W3110Rif^r^	

Then, a single clone of TC-resistant transconjugant was selected for each donor strain and analyzed by PCR with MOBH1_traI062-1F and MOBH1_traI062-2R primers. The *traI* gene was detected in five transconjugants whose donors were 04Ya001, 04Ya007, 04Ya016, 04Ya090, and 04Ya265, but not in the transconjugant whose donor was 04Ya108 (Table 5). Nucleotide sequence analysis of the PCR products revealed that the *traI* sequences derived from the transconjugants whose donors were 04Ya001, 04Ya016, 04Ya090, and 04Ya265 were identical to that of pAQU1. On the other hand, the *traI* sequences of transconjugant TJ007W1 was identical to that of its donor 04Ya007, with 97% identity to ICE*Vch*Mex.

**Table 5:** **Detection of *traI* and other antimicrobial resistance genes in donors and transconjugants by PCR**.

	Relaxase^*^		**Antimicrobial resistance genes**						
**Strain**	***traI* (pAQU1)**	***traI* (ICE*Vch*Mex)**	***tet*(B)**	***tet*(M)**	***bla*_CARB-9_-like**	***mph*(A)-like**	***mef*(A)-like**	***sul*2**	***flo*R**
04Ya001	+	−	+	+	−	−	−	−	−
TJ001	+	−	+	+	−	−	−	−	ND
04Ya007	−	+	+	+	+	+	+	+	−
TJ007	−	+	−	−	−	−	−	−	ND
04Ya016	+	−	+	+	−	−	−	−	−
TJ016	+	−	+	+	−	−	−	−	ND
04Ya090	+	−	+	+	−	−	−	−	−
TJ090	+	−	+	+	−	−	−	−	ND
04Ya108	−	−	+	+	+	+	+	+	−
TJ108	−	−	+	+	+	+	+	+	ND
04Ya265	+	−	+	+	+	+	+	+	−
TJ265	+	−	+	+	+	+	+	+	ND
04Ya311	+	−	+	+	+	+	+	+	+
TJW2	+	−	+	+	+	+	+	+	+
W3110	−	−	−		−	−	−	−	−

* By the identification of the nucleotide sequence of amplicons obtained by PCR using MOBH1_traI062-1F and MOBH1_traI062-2R.

To examine whether *tet*(M) genes in the transconjugants were also plasmid-borne, PFGE followed by Southern hybridization by using the *tet*(M) probe was performed. For transconjugants TJ001W1, TJ016W1, TJ090W1, and TJ265W1, 150 to 350-kbp long extrachromosomal DNA reactive with the *tet*(M) probe was detected similar to their corresponding donors (Figure 1A, lanes 7–9 and 11). The *traI*-pAQU probe or rep-pAQU1 probe generated signals at the same positions as *tet*(M) (Figures 1B,C, lanes 7–9 and 11). For transconjugant TJ108W1, *tet*(M) hybridization signal was detected at the position of chromosomal DNA (Figure 1A, lane 10) although its donor had plasmid-borne *tet*(M) (Figure 1A, lane 4). Although transconjugant TJ007W1 was TC-resistant, no *tet*(M) signal was detected (Figure 2A, lane 2), while the *traI*-ICE*Vch*Mex probe hybridized with the chromosomal DNA (Figure 2B, lane 2).

These results strongly suggested that the *tet*(M)-bearing pAQU1-like plasmids could be conjugatively transferred from marine bacteria to *E*. *coli*, conferring TC resistance. The mechanism by which *tet*(M) gene was transferred from 04Ya108 to TJ108W1 is unclear.

### Transfer of other ARGs associated with conjugative transfer of the pAQU1-like plasmids

PCR analysis revealed that 04Ya001, 04Ya016, 04Ya090, and 04Ya265 had *tet*(B) in addition to *tet*(M) (Table 5). Strain 04Ya265 was shown by PCR to have other ARGs, such as *sul*2, *bla*_CARB−9_-like, *mph*(A)-like and *mef*(A)-like (Table 5). Generally, all of the ARGs were transferred to the transconjugants when these strains, capable of conjugative transfer of the pAQU1-like plasmids, were used as donors (Table 5).

Incidentally, both of 04Ya007 and 04Ya108 without the pAQU1-like plasmid were shown by PCR to have all of the ARGs mentioned above (Table 5). While 04Ya007, which harbored ICE*Vch*Mex-like MGE, transferred none of these ARGs, 04Ya108 with unclassified plasmid transferred all of them (Table 5).

### Self-transferability of the pAQU1-like plasmids

To investigate whether the pAQU1-like plasmids are self-transferable, additional filter mating experiments were performed by using the transconjugants as donors and *E. coli* W3110Rif^r^ as a donor. TJ001W1, TJ090W1 and TJ265W1, which had the *tet*(M)-bearing pAQU1-like plasmid, transferred TC resistance at the frequencies (3.90 × 10^−5^ to 1.08 × 10^−4^) comparable to that of the positive control TJ311W2 (Table 4). Although TJ016W1 also had a *tet*(M)-bearing pAQU1-like plasmid, it was unable to transfer TC resistance at a detectable level.

We also tested transconjugants TJ007W1 and TJ108W1 which did not harbor the pAQU1-like plasmid. Ten times higher frequency (1.01 × 10^−3^) of TC resistance transfer was observed for TJ007W1 which had no detectable *tet*(M), whereas the transfer frequency of TJ108W1 was below the detection limit (Table 4).

### Characterization of a novel pAQU1-like plasmid, pAQU2

**Figure 3:**
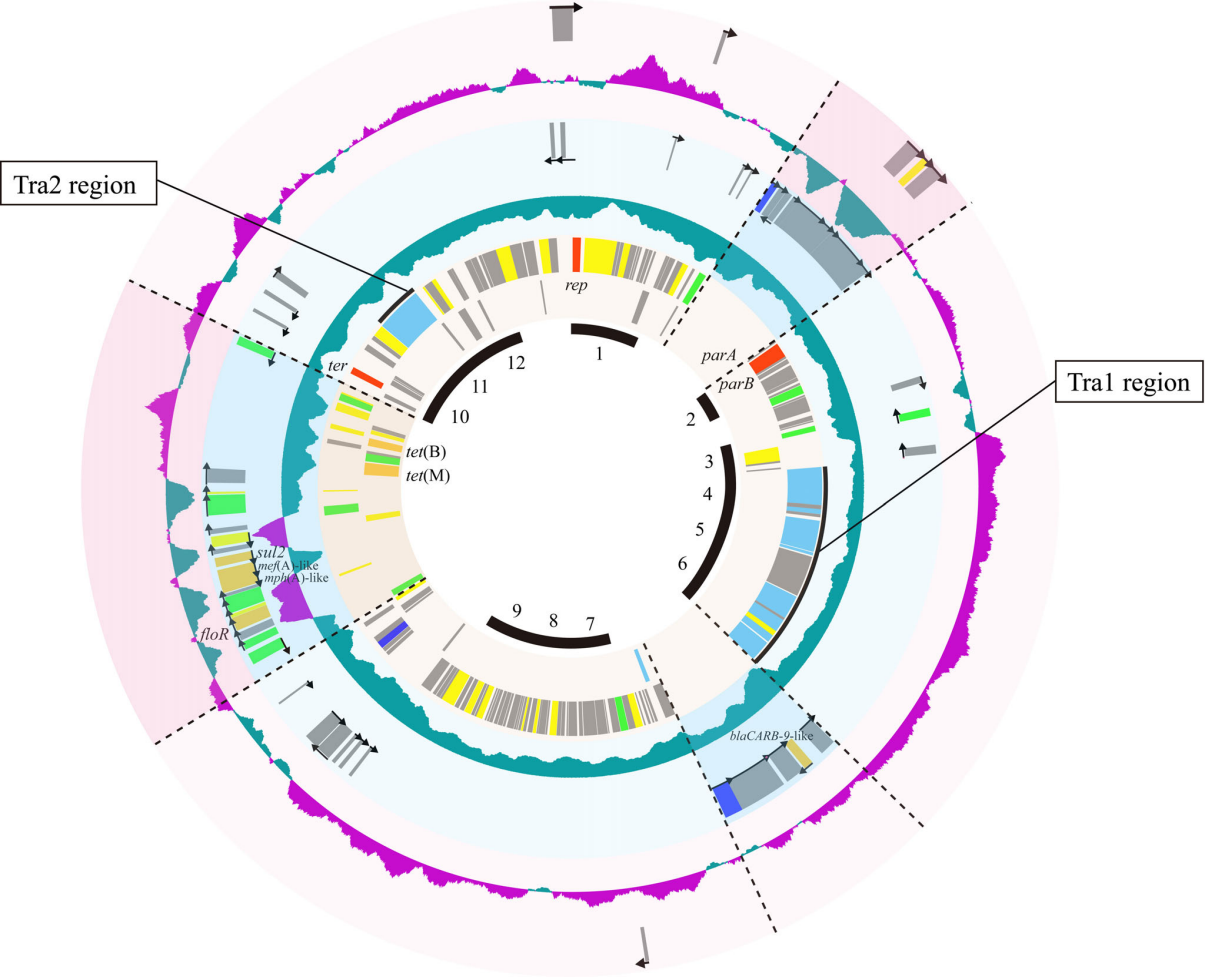
**Circular representation of pAQU1 backbone and specific genes for pAQU2 and pAQU1, respectively**. Circles from inside to outside: 1, Putative pAQU back bone regions, 2 and 3, shared CDSs between pAQU1 and pAQU2; 5 and 6, CDSs specific for pAQU1; and 8 and 9, CDSs specific for pAQU2. Each of CDSs inside and outside of the circle coded counter-clockwise and clockwise, respectively. The 4th and 7th circle indicates GC content of pAQU1 and pAQU2 where purple shows upper GC value above the center line and green shows lower GC value below the center line. Putative functions of the products of the CDSs are indicated in color: red, replication, partition and termination; light blue, conjugative transfer; blue, integration; green, transposition; orange, antibiotic resistance; yellow, other functions and gray, unknown function.

As shown above, strains 04Ya001, 04Ya016, 04Ya090, and 04Ya265 and their transconjugants, TJ001W1, TJ016W1, TJ090W1, and TJ265W1, had a pAQU1-like plasmid. Among these, we chose the plasmid in TJ090W1, which was shown to be self-transferable, and determined its entire nucleotide sequence. The novel plasmid, which we designated as pAQU2, was 160,406-bp long and contained 195 predicted coding sequences (CDSs). The CDSs were asymmetrically distributed; i.e., 163 CDSs were oriented clockwise, whereas only 32 CDSs were oriented counterclockwise (Figure 3).

Comparison of the pAQU1 and pAQU2 sequences indicated that the regions containing the genes required for basic plasmid functions, such as replication, partition and conjugative transfer, were highly conserved between the two plasmids. Thus, it was suggested that these conserved regions, which totally sum up to ~100 kbps, might constitute the backbone structure common to the pAQU1-like plasmids. In our previous study (Nonaka et al., 2012), pAQU1 was shown to have a high G+C content region with a cluster of ARGs and transposases. In pAQU2, a region with a cluster of ARGs and transposases were also found at the corresponding position, but with a low G+C content (Figure 3).

### pAQU group plasmids characterized by a common backbone structure

To test whether the backbone structure shared by pAQU1 and pAQU2 also exists in the other pAQU1-like plasmids, we characterized the donor strains 04Ya001, 04Ya016, 04Ya090, and 04Ya265 and their transconjugants by PCR with 12 sets of primers covering the entire backbone structure. The results showed that the backbone structure was maintained with no apparent deletion in the pAQU1-like plasmids harbored in all of the donors and the transconjugants examined (Table 6). Additional PCR analysis with the primers specific to some of the genes (*rep, traA2, traB, traC, traF, traN* and *traL*) in the backbone structure revealed that these genes were also retained in all of the donors and the transconjugants examined (Table 6). These results indicated that the backbone structure common to pAQU1 and pAQU2 existed in all of the other pAQU1-like plasmids examined. Therefore, there may be a novel group (pAQU group) of plasmids characterized by this backbone structure.

**Table 6:** **Detection of the conserved region (pAQU backbone) and *tra* genes specific for pAQU1 and ICE*Vch*Mex in donors and transconjugants by PCR**.

	**Region number**												**Representative genes required for conjugative transfer**									
													**pAQU1**							**ICE*Vch*Mex**		
**Strain**	**1**	**2**	**3**	**4**	**5**	**6**	**7**	**8**	**9**	**10**	**11**	**12**	***rep***	***traA2***	***traB***	***traC***	***traF***	***traN***	***traL***	***int***	***traN***	***traF***
04Ya001	+	+	+	+	+	+	+	+	+	−	+	+	+	+	+	+	+	+	+	−	−	ND
TJ001	+	+	+	+	+	+	+	+	+	−	+	+	+	+	+	+	+	+	+	−	−	ND
04Ya007	+	+	^*^	−	+	−	−	−	+	+	+	+	+	−	−	−	+	−	−	+	+	+
TJ007	−	−	−	−	−	−	−	−	−	−	−	−	−	−	−	−	−	−	−	+	+	+
04Ya016	+	+	+	+	+	+	+	+	+	+	+	+	+	+	+	+	+	+	+	−	−	ND
TJ016	+	+	+	+	+	+	+	+	+	+	+	+	+	+	+	+	+	+	+	−	−	ND
04Ya090	+	+	+	+	+	+	+	+	+	+	+	+	+	+	+	+	+	+	+	−	−	ND
TJ090	+	+	+	+	+	+	+	+	+	+	+	+	+	+	+	+	+	+	+	−	−	ND
04Ya108	−	−	−	−	−	−	−	−	−	−	−	−	−	−	−	−	−	−	−	−	−	ND
TJ108	−	−	−	−	−	−	−	−	−	−	−	−	−	−	−	−	−	−	−	−	−	−
04Ya265	+	+	+	+	+	+	+	+	+	+	+	+	+	+	+	+	+	+	+	−	−	ND
TJ265	+	+	+	+	+	+	+	+	+	+	+	+	+	+	+	+	+	+	+	−	−	ND
04Ya311	+	+	+	+	+	+	+	+	+	+	+	+	+	+	+	+	+	+	+	−	−	−
TJ311	+	+	+	+	+	+	+	+	+	+	+	+	+	+	+	+	+	+	+	−	−	−
W3110	−	−	−	−	−	−	−	−	−	−	−	−	−	−	−	−	−	−	−	−	−	−

* Amplicon sizes were smaller than expected sizes.

Interestingly, strain 04Ya007 in which ICE*Vch*Mex-like *traI*, but not pAQU1-like *traI*, was shown by PCR to harbor part of the pAQU backbone structure and some backbone-specific genes (Table 6). In addition, Southern hybridization with the *rep*-pAQU1 probe generated a band corresponding to the *tet*(M)-bearing plasmid in this strain (Figure 2A, lane 1, Figure 2C, lane 1). Therefore, it is possible that 04Ya007 carry plasmid similar to that of the pAQU group although the plasmid did not appear to be transferred to TJ007W1 (Figures 2A,C lane 2 and Table 6). Neither the pAQU backbone structure nor the backbone-specific genes were detected in 04Ya108 or its transconjugant (Table 6).

## Discussion

Recently, we discovered a novel *tet*(M)-bearing plasmid pAQU1 (204,052 bp) whose TraI primary structure was similar to that of the MOB_H12_ MGE of the MOB_H_ family and proposed that pAQU1 might play a role in dissemination of *tet*(M) and other ARGs in the marine environment (Nonaka et al., 2012). In this study, in order to investigate this possibility, we first designed degenerate PCR primers for detecting *traI* gene of the MOB_H12_ MGEs including pAQU1. With these *traI* primers, we could rapidly identify the bacterial strains which have pAQU1-like plasmids or IncA/C plasmids or SXT/R391 ICEs. Compared with the filter mating usually used for identifying the strains with transferable MGEs, the *traI*-PCR developed in this study may serve as a specific screening for the MOB_H12_ MGE-harboring bacterial strains that are frequently found in the marine environment (Welch et al., 2007; McIntosh et al., 2008; Fricke et al., 2009; Nonaka et al., 2012). The filter mating requires an appropriate recipient strain with known markers to distinguish from donor and the experiment is time consuming. On the other hand, the primers for *traI*- PCR could rapidly identify the sequence of pAQU1-like plasmids or Inc A/C plasmids or SXT/R391 ICEs.

With the *traI*-PCR method, we examined 83 strains of *tet*(M)-positive marine bacteria and found 4 *Vibrio* spp. strains (04Ya001, 04Ya016, 04Ya090, and 04Ya265) that harbored the pAQU1-like plasmids. PFGE and following Southern hybridization showed that the plasmids were 150–350 kb long and carried *tet*(M). It was also shown that the plasmids could conjugatively be transferred to *E. coli* at the frequencies of 10^−5^ to 10^−4^, generating TC-resistant transconjugants. From three (TJ001W1, TJ090W1, and TJ265W1) of the four transconjugants, the pAQU1-like plasmids were further transferred to another strain of *E. coli* at comparable frequencies, suggesting that the plasmids were self-transferable.

The entire nucleotide sequence of a *tet*(M)-bearing pAQU1-like plasmid, pAQU2, originally possessed by 04Ya090 isolated from marine sediment was determined and compared with that of pAQU1. It was shown that pAQU2 shared conserved regions, which contained the genes required for plasmid replication, partition and transfer, with pAQU1. These regions, which totally sum up to ~100 kbp, were defined as the pAQU backbone structure. The other pAQU1-like plasmids harbored in 04Ya001, 04Ya016, and 04Ya265 also shared the pAQU backbone, suggesting that these plasmids together with pAQU1 and pAQU2 could be categorized as the “pAQU group.”

In addition to *tet*(M), multiple ARGs [*tet*(B), *sul2, flo*R and genes similar to *bla*_CARB−9_, *mph*(A) and *mef*(A)] were accumulated upstream of the Tra2 region in pAQU1 where multiple transposase genes were localized, as previously described (Nonaka et al., 2012). Multiple, but fewer, ARGs were also found in pAQU2 in the corresponding region. Therefore, it was suggested that the ARGs may have been introduced through transposon-mediated DNA insertion events into the backbone structure of a prototypic plasmid. Insertion of ARGs upstream of the Tra2 region has also been reported for IncA/C plasmids (Welch et al., 2007; Fricke et al., 2009; Fernandez-Alarcon et al., 2011). Also, arrangement of *tra* genes in the pAQU backbone was quite similar to that in IncA/C plasmids and SXT/R391 ICEs, suggesting that these MGEs shared synteny. These observations are compatible with the previous study showing a close evolutionary relationship among pAQU1, IncA/C and SXT/R391 ICEs (Nonaka et al., 2012). It is possible that these MGEs had a common ancestor and evolved through acquisition of different replication systems.

Among 83 bacterial strains examined in this study, six strains were able to conjugatively confer TC resistance upon *E. coli*, of which four strains were shown to harbor the pAQU group plasmid. Therefore, it is possible that the pAQU group plasmid played a significant role in dissemination of *tet*(M) in the aquaculture site. In addition to *tet*(M), most of the ARGs carried on pAQU1 were also found in the four strains, and all of them were transmitted to *E. coli* in association with transfer of the plasmid. Therefore, it is suggested that all of the pAQU group plasmids found in this study carried not only *tet*(M) but also other ARGs. The bacterial strains found to harbor the pAQU group plasmids were isolated from the same aquaculture site, but in different times, suggesting that the pAQU group plasmids were distributed and maintained in the aquaculture environment contributing to dissemination of *tet*(M) and other ARGs among marine bacteria.

Of six bacterial strains that were able to confer TC resistance on *E. coli*, two strains did not harbor pAQU group plasmid. One of them, marine sediment strain 04Ya007 which belonged to *Shewanella* sp. and was able to transfer TC resistance at a high frequency, was shown by nucleotide sequencing of the PCR-amplified *traI* gene and Southern hybridization to have chromosomal ICE*Vch*Mex-related MGE instead. In the transconjugant obtained by filter mating by using 04Ya007 as a donor, chromosomal *traI*, as well as three other essential genes of ICE*Vch*Mex, was detected, indicating the transfer of chromosomal ICE*Vch*Mex-related MGE of 04Ya007 to the chromosome of the recipient *E. coli*. Strain 04Ya007 also had partial sequence characteristic to the pAQU backbone and plasmid-borne ARGs including *tet*(M). However, none of these pAQU group-related sequences were detected in the transconjugant. While the original ICE*Vch*Mex was reported to have no ARGs (Burrus et al., 2006), other SXT/R391 ICEs in the same group were shown to have *tet*A and *tet*R (Wozniak et al., 2009). Therefore, it is possible that the TC resistance of the transconjugant was due to an ARG(s) other than *tet*(M) and *tet*(B) that was transferred in an ICE*Vch*Mex-related MGE-dependent manner, while the *tet*(M)-bearing plasmid with the partial pAQU backbone structure had suffered from the mutation which caused loss of the transferability. Further characterization of ICE*Vch*Mex-like MGE and the plasmid in this strain is necessary to test this possibility.

The remaining strain, 04Ya108 isolated from marine sediment, which conferred TC resistance on *E. coli* at a very low frequency, appeared to have *tet*(M)-bearing plasmid of non-pAQU group, and the transconjugant was shown to have chromosome-encoded *tet*(M). Although chromosomal integration of conjugatively transferred plasmid has been reported (Jaoua et al., 1990; Ka and Tiedje, 1994; Ghosh et al., 2005), additional studies are required to elucidate the mechanism by which plasmid-born *tet*(M) of 04Ya108 integrated in the chromosome of its transconjugant.

In conclusion, PCR targeting *traI* identified four marine bacterial strains that harbor the pAQU group plasmids closely related to previously described pAQU1 (Nonaka et al., 2012). The four strains were shown by filter mating to be able to serve as donors for TC resistance. The results raise a possibility that the pAQU group plasmids might mediate conjugative transfer of *tet*(M) and other ARGs in the aquaculture site and play a major role in dissemination of drug resistance among marine bacteria.

### Conflict of interest statement

The authors declare that the research was conducted in the absence of any commercial or financial relationships that could be construed as a potential conflict of interest.

## References

[B1] Aminov R. I. Garrigues-Jeanjean N. Mackie R. I. (2001). Molecular ecology of tetracycline resistance: development and validation of primers for detection of tetracycline resistance genes encoding ribosomal protection proteins. Appl. Environ. Microbiol. 67, 22–32 10.1128/aem.67.1.22-32.2001 11133424 PMC92507

[B2] Bertrand S. Huys G. Yde M. D'haene K. Tardy F. Vrints M. (2005). Detection and characterization of *tet*(M) in tetracycline-resistant *Listeria* strains from human and food-processing origins in Belgium and France. J. Med. Microbiol. 54, 1151–1156 10.1099/jmm.0.46142-0 16278428

[B3] Burrus V. Quezada-Calvillo R. Marrero J. Waldor M. K. (2006). SXT-related integrating conjugative element in New World *Vibrio cholerae*. Appl. Environ. Microbiol. 72, 3054–3057 10.1128/aem.72.4.3054-3057.2006 16598018 PMC1449008

[B4] Cabello F. C. Godfrey H. P. Tomova A. Ivanova L. Dolz H. Millanao A. (2013). Antimicrobial use in aquaculture re-examined: its relevance to antimicrobial resistance and to animal and human health. Environ. Microbiol. 15, 1917–1942 10.1111/1462-2920.12134 23711078

[B5] Chee-Sanford J. C. Aminov R. I. Krapac I. J. Garrigues-Jeanjean N. Mackie R. I. (2001). Occurrence and diversity of tetracycline resistance genes in lagoons and groundwater underlying two swine production facilities. Appl. Environ. Microbiol. 67, 1494–1502 10.1111/1462-2920.12134 11282596 PMC92760

[B6] Chopra I. Roberts M. (2001). Tetracycline antibiotics: mode of action, applications, molecular biology, and epidemiology of bacterial resistance. Microbiol. Mol. Biol. Rev. 65, 232–260 10.1128/mmbr.65.2.232-260.2001 11381101 PMC99026

[B7] Di Cesare A. Luna G. M. Vignaroli C. Pasquaroli S. Tota S. Paroncini P. (2013). Aquaculture can promote the presence and spread of antibiotic-resistant *Enterococci* in marine sediments. PLoS ONE 8:e62838 10.1371/journal.pone.0062838 23638152 PMC3637307

[B8] Di Cesare A. Vignaroli C. Luna G. M. Pasquaroli S. Biavasco F. (2012). Antibiotic-resistant enterococci in seawater and sediments from a coastal fish farm. Microb. Drug Resist. 18, 502–509 10.1089/mdr.2011.0204 22546011

[B9] Fernandez-Alarcon C. Singer R. S. Johnson T. J. (2011). Comparative genomics of multidrug resistance-encoding IncA/C plasmids from commensal and pathogenic *Escherichia coli* from multiple animal sources. PLoS ONE 6:e23415 10.1371/journal.pone.0023415 21858108 PMC3155540

[B10] Fricke W. F. Welch T. J. Mcdermott P. F. Mammel M. K. Leclerc J. E. White D. G. (2009). Comparative genomics of the IncA/C multidrug resistance plasmid family. J. Bacteriol. 191, 4750–4757 10.1128/jb.00189-09 19482926 PMC2715731

[B11] Garcillan-Barcia M. P. Francia M. V. De La Cruz F. (2009). The diversity of conjugative relaxases and its application in plasmid classification. FEMS Microbiol. Rev. 33, 657–687 10.1111/j.1574-6976.2009.00168.x 19396961

[B12] Gevers D. Danielsen M. Huys G. Swings J. (2003). Molecular characterization of *tet*(M) genes in *Lactobacillus* isolates from different types of fermented dry sausage. Appl. Environ. Microbiol. 69, 1270–1275 10.1128/AEM.69.2.1270-1275.2003 12571056 PMC143591

[B13] Ghosh S. Mahapatra N. R. Nandi S. Banerjee P. C. (2005). Integration of metal-resistant determinants from the plasmid of an *Acidocella* strain into the chromosome of *Escherichia coli* DH5α. Curr. Microbiol. 50, 28–32 10.1007/s00284-004-4370-z 15702259

[B36] Heuer H. Smalla K. (2007). Manure and sulfadiazine synergistically increased bacterial antibiotic resistance in soil over at least two months. Environ. Microbiol. 9, 657–666 10.1111/j.1462-2920.2006.01185.x 17298366

[B14] Jaoua S. Letouvet-Pawlak B. Monnier C. Guespin-Michel J. F. (1990). Mechanism of integration of the broad-host-range plasmid RP4 into the chromosome of *Myxococcus xanthus*. Plasmid 23, 183–193 10.1016/0147-619X(90)90050-M 2120716

[B15] Jones C. H. Tuckman M. Murphy E. Bradford P. A. (2006). Identification and sequence of a *tet*(M) tetracycline resistance determinant homologue in clinical isolates of *Escherichia coli*. J. Bacteriol. 188, 7151–7164 10.1128/jb.00705-06 17015654 PMC1636245

[B16] Ka J. O. Tiedje J. M. (1994). Integration and excision of a 2,4-dichlorophenoxyacetic acid-degradative plasmid in *Alcaligenes paradoxus* and evidence of its natural intergeneric transfer. J. Bacteriol. 176, 5284–5289 8071203 10.1128/jb.176.17.5284-5289.1994 PMC196712

[B17] Kijima-Tanaka M. Kawanishi M. Fukuda Y. Suzuki S. Yagyu K. (2007). Molecular diversity of *Photobacterium damselae* ssp. *piscicida* from cultured amberjacks (Seriola spp.) in Japan by pulsed-field gel electrophoresis and plasmid profiles. J. Appl. Microbiol. 103, 381–389 10.1111/j.1365-2672.2006.03257.x 17650198

[B18] Kim S. R. Nonaka L. Suzuki S. (2004). Occurrence of tetracycline resistance genes *tet*(M) and *tet*(S) in bacteria from marine aquaculture sites. FEMS Microbiol. Lett. 237, 147–156 10.1016/j.femsle.2004.06.026 15268950

[B19] Kim Y. H. Jun L. J. Park S. H. Yoon S. H. Chung J. K. Kim J. C. (2007). Prevalence of *tet*(B) and *tet*(M) genes among tetracycline-resistant *Vibrio* spp. in the aquatic environments of Korea. Dis. Aquat. Organ. 75, 209–216 10.3354/dao075209 17629115

[B20] Larkin M. A. Blackshields G. Brown N. P. Chenna R. McGettigan P. A. McWilliam H. (2007). Clustal W and Clustal X version 2.0. Bioinformatics 23, 2947–2948 10.1093/bioinformatics/btm404 17846036

[B21] McIntosh D. Cunningham M. Ji B. Fekete F. A. Parry E. M. Clark S. E. (2008). Transferable, multiple antibiotic and mercury resistance in Atlantic Canadian isolates of *Aeromonas salmonicida* subsp. *salmonicida* is associated with carriage of an IncA/C plasmid similar to the *Salmonella enterica* plasmid pSN254. J. Antimicrob. Chemother. 61, 1221–1228 10.1093/jac/dkn123 18375380 PMC2902851

[B22] Ministry of Agriculture, Forestry and Fisheries, Japan. (2011). Protocols for the Use of Antimicrobial Agents in Aquaculture (version 24) (in Japanese).

[B23] Neela A. F. Nonaka L. Rahman M. H. Suzuki S. (2009). Transfer of the chromosomally encoded tetracycline resistance gene *tet*(M) from marine bacteria to *Escherichia coli* and *Enterococcus faecalis*. World J. Microbiol. Biotechnol. 25, 1095–1101 10.1007/s11274-009-0004-8

[B24] Nikolakopoulou T. L. Giannoutsou E. P. Karabatsou A. A. Karagouni A. D. (2008). Prevalence of tetracycline resistance genes in Greek seawater habitats. J. Microbiol. 46, 633–640 10.1007/s12275-008-0080-8 19107391

[B25] Noguchi H. Taniguchi T. Itoh T. (2008). MetaGeneAnnotator: detecting species-specific patterns of ribosomal binding site for precise gene prediction in anonymous prokaryotic and phage genomes. DNA Res. 15, 387–396 10.1093/dnares/dsn027 18940874 PMC2608843

[B26] Nonaka L. Ikeno K. Suzuki S. (2007). Distribution of tetracycline resistance gene, *tet*(M), in Gram-positive and Gram-negative bacteria isolated from sediment and seawater at a coastal aquaculture site in Japan. Microbes Environ. 22, 355–364 10.1264/jsme2.22.355

[B27] Nonaka L. Maruyama F. Miyamoto M. Miyakoshi M. Kurokawa K. Masuda M. (2012). Novel conjugative transferable multiple drug resistance plasmid pAQU1 from *Photobacterium damselae* subsp. *damselae* isolated from marine aquaculture environment. Microbes Environ. 27, 263–272 10.1264/jsme2.ME11338 22446310 PMC4036041

[B28] Paigen K. (1966) Phenomenon of transient repression in *Escherichia coli*. J. Bacteriol. 91, 1201–1209 5326097 10.1128/jb.91.3.1201-1209.1966 PMC316014

[B29] Roberts M. C. (2005). Update on acquired tetracycline resistance genes. FEMS Microbiol. Lett. 245, 195–203 10.1016/j.femsle.2005.02.034 15837373

[B30] Sambrook J. Russell D. W. (2001). Molecular Cloning: A Laboratory Manual, 3rd Edn. New York, NY: Cold Spring Harbor Laboratory Press

[B31] Seyfried E. E. Newton R. J. Rubert K. F. T. Pedersen J. A. Mcmahon K. D. (2010). Occurrence of tetracycline resistance genes in aquaculture facilities with varying use of oxytetracycline. Microb. Ecol. 59, 799–807 10.1007/s00248-009-9624-7 20217406 PMC4066850

[B32] Smillie C. Garcillan-Barcia M. P. Francia M. V. Rocha E. P. De La Cruz F. (2010). Mobility of plasmids. Microbiol. Mol. Biol. Rev. 74, 434–452 10.1128/mmbr.00020-10 20805406 PMC2937521

[B33] Thompson J. D. Higgins D. G. Gibson T. J. (1994). CLUSTAL W: improving the sensitivity of progressive multiple sequence alignment through sequence weighting, position-specific gap penalties and weight matrix choice. Nucleic Acids Res. 22, 4673–4680 10.1093/nar/22.22.4673 7984417 PMC308517

[B34] Welch T. J. Fricke W. F. Mcdermott P. F. White D. G. Rosso M. L. Rasko D. A. (2007). Multiple antimicrobial resistance in plague: an emerging public health risk. PLoS ONE 2:e309 10.1371/journal.pone.0000309 17375195 PMC1819562

[B35] Wozniak R. A. Fouts D. E. Spagnoletti M. Colombo M. M. Ceccarelli D. Garriss G. (2009). Comparative ICE genomics: insights into the evolution of the SXT/R391 family of ICEs. PLoS Genet 5:e1000786 10.1371/journal.pgen.1000786 20041216 PMC2791158

