# Supplementary material for: Various pAQU plasmids possibly contribute to disseminate tetracycline resistance gene tet(M) among marine bacterial community
Source: Front Microbiol. 2014 May 12;5:152. doi: 10.3389/fmicb.2014.00152 (PMC4026752; doi:10.3389/fmicb.2014.00152)
Supplement: Supplementary file 1 [file DataSheet1.DOC]

Supplementary **Table 1**.

Strains used in this study.

| Strain | Genus or species | Samling site* | Origin* | Detection of *tet*(M) by PCR* | Isolation Date** | Concentration of OTC（μg/ml） in the Marine broth plate used for isolation* |
| --- | --- | --- | --- | --- | --- | --- |
| 04Ya001 | *Vibrio* sp. | 1 | Sediment (0-3 cm) | + | April 27 | 480 |
| 04Ya004 | *Vibrio* sp. | 1 | Sediment (0-3 cm) | + | April 27 | 480 |
| 04Ya007 | *Shewanella* sp. | 1 | Sediment (0-3 cm) | + | April 27 | 480 |
| 04Ya016 | *Vibrio* sp. | 1 | Sediment (0-3 cm) | + | April 27 | 480 |
| 04Ya030 | *Vibrio* sp. | 2 | Sediment (0-3 cm) | + | April 27 | 480 |
| 04Ya033 | *Vibrio* sp. | 2 | Sediment (0-3 cm) | + | April 27 | 240 |
| 04Ya045 | *Vibrio* sp. | 3 | Sediment (0-3 cm) | + | April 27 | 480 |
| 04Ya050 | *Vibrio* sp. | 3 | Sediment (0-3 cm) | + | April 27 | 480 |
| 04Ya057 | *Vibrio* sp. | 3 | Sediment (3-6 cm) | + | April 27 | 480 |
| 04Ya059 | *Vibrio* sp. | 3 | Sediment (3-6 cm) | + | April 27 | 240 |
| 04Ya068 | *Vibrio* sp. | 1 | Seawater | + | April 27 | 240 |
| 04Ya086 | *Vibrio* sp. | 1 | Sediment (0-3 cm) | + | May 21 | 480 |
| 04Ya090 | *Vibrio* sp. | 1 | Sediment (0-3 cm) | + | May 21 | 480 |
| 04Ya094 | *Vibrio* sp. | 1 | Sediment (0-3 cm) | + | May 21 | 480 |
| 04Ya100 | *Vibrio* sp. | 1 | Sediment (3-6 cm) | + | May 21 | 480 |
| 04Ya101 | *Vibrio* sp. | 1 | Sediment (3-6 cm) | + | May 21 | 480 |
| 04Ya103 | *Vibrio* sp. | 1 | Sediment (3-6 cm) | + | May 21 | 480 |
| 04Ya108 | *Vibrio* sp. | 1 | Sediment (3-6 cm) | + | May 21 | 480 |
| 04Ya110 | *Vibrio* sp. | 1 | Sediment (3-6 cm) | + | May 21 | 480 |
| 04Ya111 | *Vibrio* sp. | 2 | Sediment (0-3 cm) | + | May 21 | 480 |
| 04Ya112 | *Shewanella* sp. | 2 | Sediment (0-3 cm) | + | May 21 | 480 |
| 04Ya115 | *Vibrio* sp. | 2 | Sediment (0-3 cm) | + | May 21 | 480 |
| 04Ya117 | *Vibrio* sp. | 2 | Sediment (0-3 cm) | + | May 21 | 480 |
| 04Ya121 | *Vibrio* sp. | 2 | Sediment (0-3 cm) | + | May 21 | 480 |
| 04Ya123 | *Vibrio* sp. | 2 | Sediment (0-3 cm) | + | May 21 | 480 |
| 04Ya129 | *Vibrio* sp. | 2 | Sediment (3-6 cm) | + | May 21 | 480 |
| 04Ya137 | *Vibrio* sp. | 2 | Sediment (3-6 cm) | + | May 21 | 480 |
| 04Ya142 | *Vibrio* sp. | 2 | Sediment (3-6 cm) | + | May 21 | 480 |
| 04Ya148 | *Vibrio* sp. | 3 | Sediment (0-3 cm) | + | May 21 | 480 |
| 04Ya150 | *Vibrio* sp. | 3 | Sediment (0-3 cm) | + | May 21 | 480 |
| 04Ya155 | *Vibrio* sp. | 3 | Sediment (3-6 cm) | + | May 21 | 480 |
| 04Ya181 | *Shewanella* sp. | 1 | Sediment (0-3 cm) | + | June 25 | 480 |
| 04Ya186 | *Vibrio* sp. | 1 | Sediment (0-3 cm) | + | June 25 | 240 |
| 04Ya192 | *Sporosarcina* sp. | 1 | Sediment (3-6 cm) | + | June 25 | 240 |
| 04Ya208 | *Vibrio* sp. | 3 | Sediment (0-3 cm) | + | June 25 | 240 |
| 04Ya209 | *Bachillus* sp. | 3 | Sediment (0-3 cm) | + | June 25 | 240 |
| 04Ya228 | *Vibrio* sp. | 1 | Sediment (0-3 cm) | + | Sep 24 | 480 |
| 04Ya230 | *Vibrio* sp. | 1 | Sediment (0-3 cm) | + | Sep 24 | 480 |
| 04Ya231 | *Photobacterium* sp. | 1 | Sediment (0-3 cm) | + | Sep 24 | 480 |
| 04Ya232 | *Vibrio* sp. | 1 | Sediment (0-3 cm) | + | Sep 24 | 480 |
| 04Ya233 | *Photobacterium* sp. | 1 | Sediment (0-3 cm) | + | Sep 24 | 480 |
| 04Ya234 | *Vibrio* sp. | 1 | Sediment (0-3 cm) | + | Sep 24 | 480 |
| 04Ya238 | Bachillus sp. | 1 | Sediment (3-6 cm) | + | Sep 24 | 480 |
| 04Ya239 | *Vibrio* sp. | 1 | Sediment (3-6 cm) | + | Sep 24 | 480 |
| 04Ya244 | *Vibrio* sp. | 2 | Sediment (0-3 cm) | + | Sep 24 | 480 |
| 04Ya245 | *Photobacterium* sp. | 2 | Sediment (0-3 cm) | + | Sep 24 | 480 |
| 04Ya246 | *Vibrio* sp. | 2 | Sediment (0-3 cm) | + | Sep 24 | 480 |
| 04Ya247 | *Vibrio* sp. | 2 | Sediment (0-3 cm) | + | Sep 24 | 480 |
| 04Ya248 | *Vibrio* sp. | 2 | Sediment (0-3 cm) | + | Sep 24 | 480 |
| 04Ya249 | *Vibrio* sp. | 2 | Sediment (0-3 cm) | + | Sep 24 | 480 |
| 04Ya252 | *Bachillus* sp. | 2 | Sediment (3-6 cm) | + | Sep 24 | 480 |
| 04Ya253 | *Lactobachillus* sp. | 2 | Sediment (3-6 cm) | + | Sep 24 | 240 |
| 04Ya254 | *Paenibacillus* sp. | 2 | Sediment (3-6 cm) | + | Sep 24 | 240 |
| 04Ya255 | *Vibrio* sp. | 2 | Sediment (3-6 cm) | + | Sep 24 | 240 |
| 04Ya257 | *Bacillus* sp. | 2 | Sediment (3-6 cm) | + | Sep 24 | 240 |
| 04Ya258 | *Vibrio* sp. | 3 | Sediment (0-3 cm) | + | Sep 24 | 240 |
| 04Ya261 | *Vibrio* sp. | 3 | Sediment (3-6 cm) | + | Sep 24 | 240 |
| 04Ya262 | *Vibrio* sp. | 3 | Sediment (3-6 cm) | + | Sep 24 | 240 |
| 04Ya264 | *Vibrio* sp. | 3 | Sediment (3-6 cm) | + | Sep 24 | 240 |
| 04Ya265 | *Vibrio* sp. | 4 | Sediment (0-3 cm) | + | Sep 24 | 480 |
| 04Ya266 | *Vibrio* sp. | 4 | Sediment (0-3 cm) | + | Sep 24 | 480 |
| 04Ya267 | *Vibrio* sp. | 4 | Sediment (0-3 cm) | + | Sep 24 | 480 |
| 04Ya268 | *Vibrio* sp. | 4 | Sediment (0-3 cm) | + | Sep 24 | 480 |
| 04Ya269 | *Vibrio* sp. | 4 | Sediment (0-3 cm) | + | Sep 24 | 480 |
| 04Ya275 | *Vibrio* sp. | 4 | Sediment (0-3 cm) | + | Sep 24 | 240 |
| 04Ya279 | *Vibrio* sp. | 1 | Seawater | + | Sep 24 | 480 |
| 04Ya290 | *Vibrio* sp. | 1 | Seawater | + | Sep 24 | 240 |
| 04Ya294 | *Vibrio* sp. | 2 | Seawater | + | Sep 24 | 480 |
| 04Ya296 | *Vibrio* sp. | 2 | Seawater | + | Sep 24 | 480 |
| 04Ya297 | *Vibrio* sp. | 2 | Seawater | + | Sep 24 | 480 |
| 04Ya301 | *Vibrio* sp. | 3 | Seawater | + | Sep 24 | 480 |
| 04Ya302 | *Vibrio* sp. | 3 | Seawater | + | Sep 24 | 480 |
| 04Ya303 | *Vibrio* sp. | 3 | Seawater | + | Sep 24 | 480 |
| 04Ya304 | *Vibrio* sp. | 3 | Seawater | + | Sep 24 | 480 |
| 04Ya305 | *Vibrio* sp. | 3 | Seawater | + | Sep 24 | 480 |
| 04Ya306 | *Pseudomonas* sp. | 3 | Seawater | + | Sep 24 | 480 |
| 04Ya309 | *Vibrio* sp. | 4 | Seawater | + | Sep 24 | 480 |
| 04Ya310 | *Vibrio* sp. | 4 | Seawater | + | Sep 24 | 480 |
| 04Ya311  (positive control) | *Photobacterium* *damselae* subsp. *damselae* | 4 | Seawater | + | Sep 24 | 480 |
| 04Ya312 | *Vibrio* sp. | 4 | Seawater | + | Sep 24 | 480 |
| 04Ya313 | *Vibrio* sp. | 4 | Seawater | + | Sep 24 | 480 |
| 04Ya314 | *Vibrio* sp. | 4 | Seawater | + | Sep 24 | 480 |
| 04Ya315 | *Vibrio* sp. | 4 | Seawater | + | Sep 24 | 480 |

*referred Nonaka *et al*., 2007

**Sampling was performed in 2004.
